# Systems and implementation science should be part of the COVID-19 response in low resource settings

**DOI:** 10.1186/s12916-020-01696-6

**Published:** 2020-07-15

**Authors:** Mike English, Mosa Moshabela, Jacinta Nzinga, Edwine Barasa, Benjamin Tsofa, Bruno Marchal, Margaret E Kruk

**Affiliations:** 1 Centre for Tropical Medicine and Global Health, Nuffield Department of Medicine, University of Oxford, Oxford, UK; 2 KEMRI-Wellcome Trust Research Programme, Nairobi, Kenya; 3 School of Nursing and Public Health, University of KwaZulu Natal, Durban, South Africa; 4 Centre for Geographic Medicine Research – Coast, KEMRI, Kilifi, Kenya; 5 Institute for Tropical Medicine, Antwerp, Belgium; 6 Harvard T.H. Chan School of Public Health, Harvard University, Boston, USA

## Background

The huge strategic response to COVID-19 is, put simply, the globe’s single largest and most concentrated health service redesign effort. To counter the effects of the pandemic in Low Resource Settings (LRS) the World Bank has promised to invest $160 billion into Africa over the next 15 months with bilateral donors also pledging large sums to support COVID-19 responses (e.g. USAID $775 million, UK-AID £200 million). Over 60 LRS governments had applied for World Bank funding by 19th June 2020 with plans to spend huge sums on health system strengthening at unprecedented speed [1]. UN agencies and a multitude of technical assistance organisations are reorienting their work to support countries in areas spanning guideline dissemination, training, improving information systems and equipment and consumable production, procurement and supply chain management amongst others. Some LRS governments are planning emergency recruitment to address critical workforce deficits. It is imperative, therefore, that we use the COVID-19 moment to optimise learning about how to transform health service delivery to benefit population health.

## The research response

A recently published ‘call to action’ to accelerate clinical research around COVID-19 in LRS [2] acknowledged the need for such learning and that COVID-19, and the response to it, might disrupt weak health systems resulting in harm directly and through unintended consequences. It recognised that clinical research products might never benefit LRS populations because of failures to adopt, afford or implement them. Overwhelmingly, however, this call to action was devoted to research on disease epidemiology, development and trials of clinical therapeutics and vaccines. Vaccines and therapies are critical—as they are for a host of diseases that plague LRS—and so furiously paced research to identify them must be central to the research endeavour. However, although this prior call to action stated that ‘evaluations of affordable and implementable interventions of all types—behavioural, organisational, medical, and supportive—are a priority’, scant attention seems devoted to this agenda even in the published COVID-19 research priorities of WHO or the African Academy of Sciences [3, 4].

As in richer countries, perhaps the most visible sign of COVID-19 health services investments will be efforts to upgrade hospitals to cater for anticipated surges in severely ill patients. This may undermine efforts to ensure Universal Health Coverage unless such hospital strengthening is part of integrated, longer-term planning. For the clinical management of severe COVID-19, LRS may aspire to global best practice but specific country contexts should determine where in the spectrum from essential care to full-scale intensive care countries begin to focus and over time hope to land. Beyond plans or strategies, actual delivery of clinical COVID-19 services will be strongly influenced by the dynamic interplay with local contexts. In facilities, these range from physical size and layout, adequacy of basic infrastructure (e.g. power supply and adequacy of water, sanitation and hygiene that support infection control) to numbers of staff and skill mix and much in-between. But resources alone will not determine a successful response; that will depend on organisational “software” spanning governance arrangements, institutional norms, local management and culture and how they influence inter-professional, team and individual behaviours. Such factors are well-recognised in theories of effective health care organisations, in implementation science and the realm of quality improvement more broadly [5].

Ideally, we should take advantage of COVID-19 investments to transform health systems for the long term and ally this to rigorous systems and implementation research [6]. For example, the service redesign needed to treat severe COVID-19 may deliver much wider learning about the how best to provide key hospital services such as triage, emergency and critical care across a range of contexts from rural hospitals to tertiary care. The COVID-19 response is hoping to raise the bar for information and surveillance systems, introducing new technologies and changing work patterns, including task sharing between different professionals, all areas worthy of study. Important lessons may be learned about development, distribution and adoption of innovations. For example, manufacturers have turned their attention to lower cost respiratory support technologies and rapid scaling up of access to oxygen to strengthen critical care supported by new integrated supply mechanisms [7]. Understanding how these efforts are translated into effective forms of care, or whether they result in unintended harms to individuals or systems, may inform much wider efforts to strengthen and improve surgical care, maternal and neonatal care, or care for other serious adult illnesses such as acute cardiovascular disease events in LRS. Specifically, organised learning health systems are increasingly used in high-income countries for the purposes of such evaluations but are rare in LRS although some examples are emerging [6, 8].

## Conclusion

In mounting a broad systems and implementation research response to redesigning today’s health systems, we recognise that no single theory, framework or ‘toolbox’ is suitable for all questions and settings. However, researchers should be as rigorous as possible in designing, conducting and reporting their work with the specific purpose of generating needed inputs to local policy as well as generalizable insights. Generating transferable knowledge would be facilitated by multi-country research collaborations that share expertise, methods and priorities as WHO is promoting through the Quality of Care Network focused on peripartum care [9]. Another such initiative is the proposed QuEST Network, a follow-on body to the Lancet Global Health Commission on High Quality Health Systems. Such consortia may provide models for jointly tackling large-scale, high-priority research that supports health system reform toward high-quality, people-centred care during and after the COVID-19 pandemic. In promoting and conducting such work, there is an opportunity to build much needed capacity amongst LRS scientists and foster the continued growth of fields that the COVID-19 pandemic illustrates are key to supporting evidence-informed high-quality health systems in the long run [10].
